# Functional animal proteins activate mTOR and bind pro-inflammatory compounds

**DOI:** 10.1186/1550-2783-12-S1-P35

**Published:** 2015-09-21

**Authors:** Christopher J Detzel, Michael Q Fleming, Christopher D Warner, Abigail L Henderson, Eric M Weaver

**Affiliations:** 1 Essentia Metabolic Proteins, Ankeny, IA, USA

## Background

Protein supplementation in addition to resistance training has been shown to increase muscle hypertrophy and lean mass. Supplemental protein sources differ in amino acid composition, size, structure, and functionality. Animal derived proteins sources such as Beef Protein Isolate (BeefISO), Serum Albumin Concentrate (SuperSerum), Serum Protein Concentrate (SerumPro), whey protein isolate (WPI), and hydrolyzed Chicken Protein Isolate (MyoCHX) each have high-quality amino acid profiles. The mammalian target of rapamycin (mTOR) signal pathway is a nutrient sensor whose activation is associated with muscle protein synthesis. In this work, mTOR pathway activation was shown by Western blot to demonstrate bioavailability of protein preparations. Protein functionality was demonstrated by lipopolysaccharide (LPS) binding to prevent antigen induced inflammatory signaling. Systemic inflammation has been shown to negatively impact athletic performance, suggesting protein preparations which can stimulate muscle protein synthesis and reduce inflammation may be advantageous following resistance training.

## Methods

HEK293 cells were stimulated by protein preparations and probed for activation of the mTOR signaling pathway. Briefly, cells were serum starved for 24 hours followed by addition of protein stimulants normalized in protein content. Cells were exposed to 5% proteins solutions for 2, 10, 30, and 60 min after which cellular proteins were harvested for Western blot analysis. Activation of mTOR was monitored at the Ser2448 phosphorylation site. The housekeeping protein β-actin was used to normalize protein loading conditions for comparison between protein preparations. Endotoxin neutralization experiments were conducted by measuring the inflammatory response of THP-1 monocytes to lipopolysaccharide (LPS). Protein solutions (1.25% w/v) were mixed with 10 ng/mL LPS for 1 hour prior to THP-1 exposure. THP-1 and protein mixture were incubated for 24 hours followed by analysis of IL-8 inflammatory cytokine production by Bio-Plex^® ^MAGPIX™ Multiplex Reader and Bio-Plex Pro™ Assays (Bio-Rad, Hercules, CA). IL-8 inhibition was determined by comparison with a standard curve of THP-1 responses to varying concentrations of LPS.

## Results

Each of the animal protein supplements tested activated the mTOR pathway as evidenced by increased phosphorylation of mTOR compared with controls. BioBeef, BeefISO, SerumPro, WPI and MyoCHX stimulated the highest phosphorylation of mTOR at 10 min post-stimulation while SuperSerum resulted in earlier maximal stimulation at 2 min. Serum derived protein supplements (BioBeef, SerumPro, and SuperSerum) were each capable of neutralizing endotoxin as shown by a significant (p < 0.05) decrease in IL-8 inflammatory cytokine production by THP-1 monocytes when compared to addition of LPS alone. Blending of high-quality protein sources with functional serum protein supplements (SuperSerum and SerumPro) resulted in the effective inhibition of LPS-induced inflammation at an inclusion rate of SuperSerum or SerumPro as low as 5% of the total protein.

## Conclusion

Animal derived protein supplements are quickly absorbed by cells *in vitro *and efficiently activate the mTOR signaling pathway, which is associated with increased MPS. High-intensity exercise has been shown to increase inflammation in the body in part from responses to inflammatory antigens. Functional serum proteins provide high quality amino acids yet have a unique impact on immune exclusion through protein binding to inflammatory antigens resulting in anti-inflammatory benefits.

